# Case Report: Neurological brucellosis with behavioral abnormalities: a case of Brucella encephalitis with mild cognitive impairment

**DOI:** 10.3389/fmed.2026.1809313

**Published:** 2026-04-15

**Authors:** Yinlan Su, Wenjing Deng, Xueyun Wang

**Affiliations:** Department of Infectious Diseases Jining Public Health Medical Center (Jining Fourth People's Hospital), Jining, China

**Keywords:** abnormal behavior, cognitive impairment, diagnosis, encephalitis, neurological brucellosis, NGS

## Abstract

Brucellosis remains widespread in the central and western parts of China, as well as in pastoral regions like Inner Mongolia. In recent years, however, its geographic spread has expanded into certain areas of the Middle East and northern China, where *Brucella melitensis* has emerged as the dominant strain. Meanwhile, strains associated with cattle and pigs have seen a decline in prevalence. Although neurological brucellosis is relatively rare among brucellosis cases, it presents a considerable challenge due to its non-specific symptoms and diverse clinical manifestations, which often lead to misdiagnosis or delayed recognition. The most commonly reported symptoms of neurological brucellosis include headaches, along with visual disturbances, hearing impairment, confusion, sleep disorders, seizures, agitation, depression, and signs of peripheral nerve involvement. In instances of encephalitis, alterations in consciousness and focal neurological deficits are frequently observed. Furthermore, it is important to note that certain patients may display psychiatric or behavioral changes. In such instances, obtaining a comprehensive epidemiological history and conducting thorough laboratory tests are essential for an accurate diagnosis.

## 1 Introduction

Neurological brucellosis (NB), a severe complication of brucellosis, arises from Brucella infection of the nervous system. Both clinical and imaging findings are often non-specific (1–3), which, coupled with the broad range of symptoms, can significantly impede early diagnosis (3–5). A four-year study identified regional disparities in the incidence rate of brucellosis, despite the national average annual incidence being 3.0 per 100,000 (6). For patients presenting with neurological symptoms, the potential diagnosis of neurological brucellosis should be considered when Brucella pathogens are detected in any system (7, 8). This article discusses a case of neurological brucellosis in which cognitive impairment was the primary manifestation, with confirmation through cerebrospinal fluid gene sequencing. The case aims to serve as a valuable reference for clinical diagnosis and treatment, promoting early detection, reducing the risk of misdiagnosis and mistreatment, and ultimately enhancing patient outcomes.

## 2 Manuscript formatting

### 2.1 Case presentation

A 70-year-old male farmer was first admitted to our hospital on June 2, 2025, with “recurrent fever accompanied by headache for more than 6 months, and sudden confusion for 2 days.” Six months before admission, he began to experience headache, predominantly on the right side, along with low back pain, fever, fatigue, and poor appetite. At that stage, he remained fully conscious, with no bradyphrenia, lower-limb numbness, or tremor of the extremities, and did not seek medical attention because the symptoms were not considered serious. Three months later, as the back pain and headache worsened, he visited a local hospital, where a positive Brucella agglutination test (1:400) raised the suspicion of brucellosis. He was then treated with rifampin in combination with doxycycline for more than 10 days.

Two months before the present admission, the patient developed acute confusion, incoherent speech, and an inability to answer questions appropriately, after which he was transferred to our hospital. Urinalysis, blood culture, and tests for influenza virus and Epstein-Barr virus were performed, effectively excluding pyelonephritis/renal abscess, influenza, and other viral infections. Further inquiry into his epidemiological history revealed that he raised sheep at home, had direct contact with live sheep and their secretions, and had a family history of brucellosis. Cranial MRI showed cerebral arteriosclerosis, multiple cerebral infarcts and ischemic lesions, brain atrophy, and white matter degeneration. Although these neuroimaging findings were non-specific, neurobrucellosis could not be ruled out completely; intracranial neoplasm and lumbar disc disease were also taken into consideration in the differential diagnosis.

Given that both the epidemiological background and laboratory findings were consistent with brucellosis, empirical treatment with rifampin, doxycycline, ceftriaxone, and co-trimoxazole was initiated and continued for more than 20 days. The patient became afebrile, and his low back pain and headache improved, suggesting a favorable response to antimicrobial therapy. Taken together with the serological and imaging findings, a diagnosis of brucellosis was made. He was discharged on June 22, 2025. After discharge, however, he developed dry retching and vomiting while taking the prescribed medications, which markedly interfered with oral intake. He therefore discontinued the medication on his own and did not return for scheduled follow-up.

**Figure 1:**
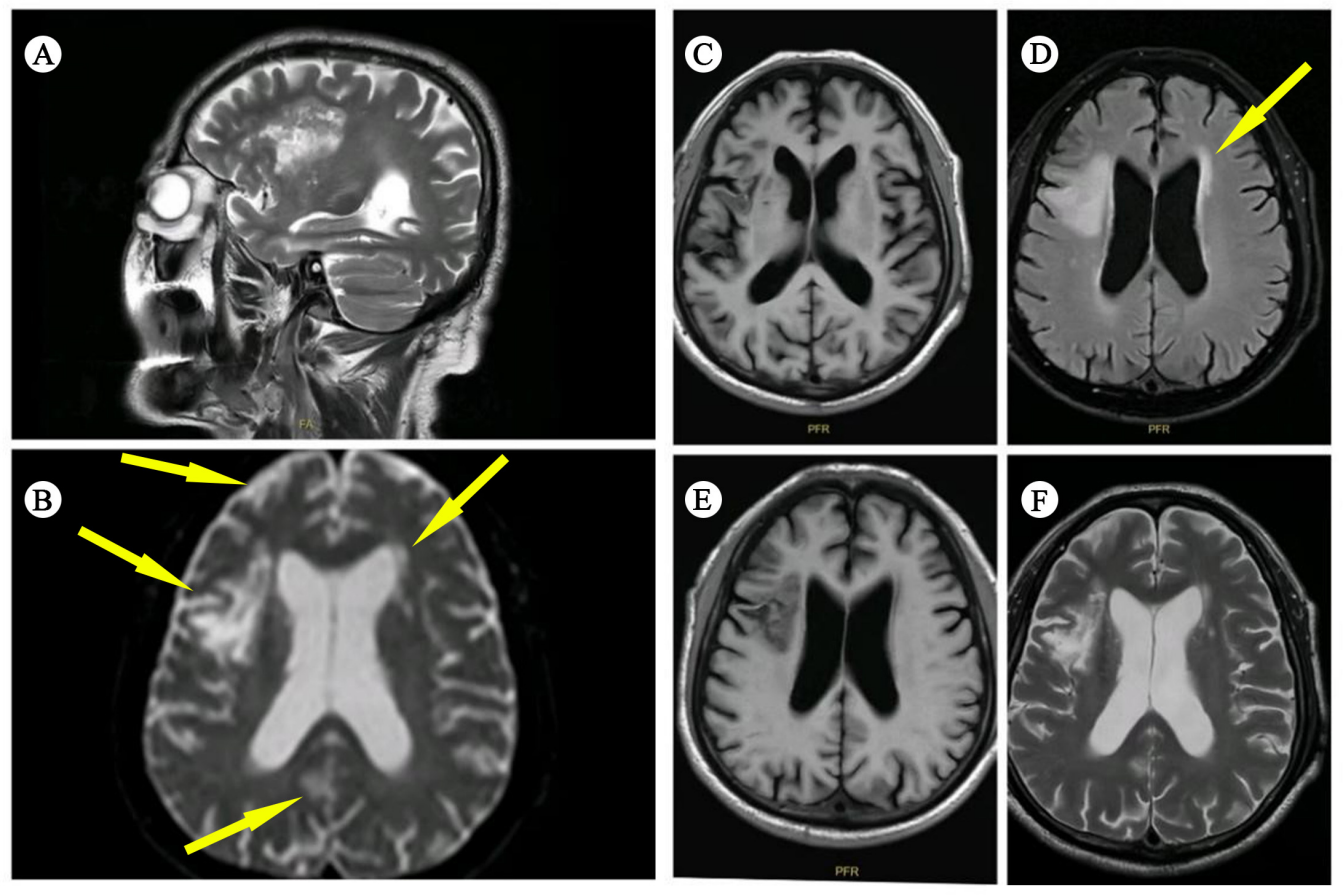
Brain magnetic resonance imaging findings. **(A)** Sagittal T2-weighted fast spin echo sequence. **(B)** Axial diffusion-weighted imaging at the ventricular level. **(C)** Axial T1-weighted fluid-attenuated inversion recovery sequences. **(D)** Axial T2-weighted fluid-attenuated inversion recovery sequence with fat suppression. **(E)** Axial T1-weighted fluid-attenuated inversion recovery sequences. **(F)** Axial T2-weighted fast spin echo sequence.

After discontinuing the medication for 20 days, the patient developed new behavioral abnormalities, including impaired consciousness, headache, and lower back pain. Neurological examination revealed signs of inattention, non-fluent aphasia, memory deficits, bilateral resting and action tremors, bradykinesia, and muscle weakness. On July 15, 2025, a cranial MRI showed multiple infarcts, ischemic lesions, brain atrophy, and signs of cerebral arteriosclerosis on MRI, (Figure 1). Compared to the initial MRI, no significant changes were observed in the lesions. On July 13, 2025, the patient's Brucella agglutination test (1:200) returned a positive result (+++). Additional laboratory findings, including ESR, liver and kidney function tests, complete blood count, electrolytes, and T-spot, revealed a low white blood cell count with neutrophil predominance, while red blood cells and platelets remained within normal limits. Given the patient's age, mental and behavioral abnormalities, and medical history, neurological brucellosis was strongly suspected. As a result, an empirical treatment regimen of rifampin, doxycycline, ceftriaxone, co-trimoxazole, and low-dose mannitol was initiated for 2 days. This treatment led to an improvement in his headache symptoms, with the antimicrobial therapy proving effective. The patient's mental status also showed significant progress, and he became more cooperative during procedures.

**Figure 2:**
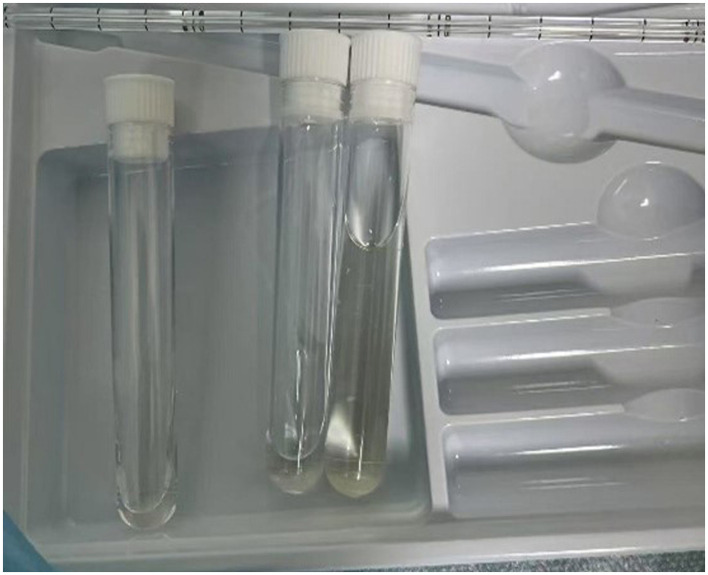
Patient cerebrospinal fluid image.

On July 17, 2025, a lumbar puncture was performed to collect cerebrospinal fluid (CSF), revealing elevated intracranial pressure of 220 cmH_2_O. The CSF was clear and pale yellow (Figure 2). Routine CSF analysis, biochemical tests, fungal examination, metagenomic next-generation sequencing (mNGS) for pathogen detection, and Brucella agglutination tests on the CSF were conducted. The results showed: positive protein, white blood cells at 88 × 10^6^/L, with 90.9% mononuclear cells and 9.1% polymorphonuclear cells, no red blood cells, chloride at 114.7 mmol/L (decreased), total protein at 134.6 mg/dL (increased), adenosine deaminase at 10.3 U/L, and glucose at 2.0564 mmol/L. No fungal hyphae or spores were observed on the fungal smear. The CSF examination revealed an increase in white blood cells, with mononuclear cells predominating (Table 1), suggesting a potential central nervous system infection. Following the initiation of empirical treatment, the patient's headache and mental status improved.

**Table 1:** Patient's cerebrospinal fluid test results.

Abbreviation	Project name	Result	Unit	Reference range	Methodology
CL	Chlorine	114.7	mmol/L	120–130	Ion electrode method
Tp-C	Total protein	134.6	mmol/L	15–45	Cresyl red molybdenum method
ADA-C	Adenosine deaminase	10.3	U/L	–	Rate method
GLU-X	Glucose	2.0564	mmol/L	–	Hexokinase method
zd	Turbidity	Clear	–	Transparent	–
C-DBDX	Protein qualitative analysis	Positive	–	–	–
bxb	White blood cells	88	106/L	0–8	–
agh	Single nucleated cell	90.9	%	–	–
dghxb	multinucleated cells	9.1	%	–	–
hxb	Red blood cells	0	106/L	–	–

**Figure 3:**
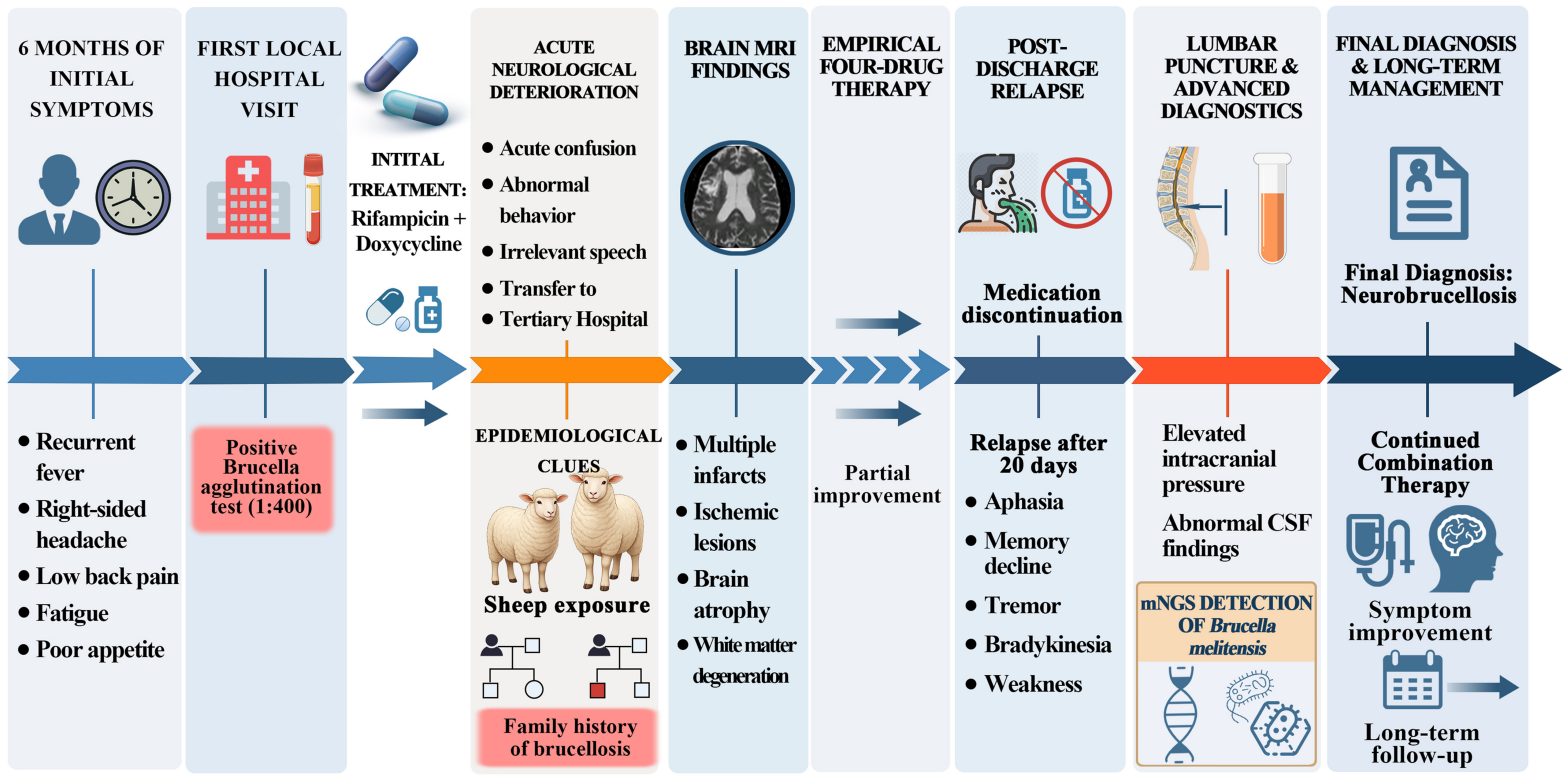
Flowchart of patient progression and treatment methods (34). Created with BioGDP.com.

On July 23, 2025, mNGS results confirmed the presence of *Brucella melitensis* (pathogenic A type) and Epstein-Barr virus (pathogenic B type) in the CSF. Consequently, mNGS was used to analyze the pathogens in the cerebrospinal fluid, identifying *Brucella* as the causative agent. After confirming the diagnosis, the four-drug antibiotic regimen was continued for more than a month. The Brucella agglutination test (1:200) remained positive throughout treatment, and the antimicrobial therapy showed partial effectiveness. Upon discharge, the patient exhibited slower responses but showed no further significant behavioral abnormalities, headaches, or other discomforts. He is currently undergoing continued antimicrobial therapy with rifampin, doxycycline, and co-trimoxazole. Over the next 6 months or longer, the patient will be monitored through serological, microbiological, and MRI imaging tests, with ongoing follow-up until full recovery (Figure 3).

### 2.2 Discussion

The patient primarily presented with fever, headache, cognitive decline, and neuropsychiatric symptoms, demonstrating a clinical course that progressed from a systemic infection to involvement of the central nervous system. Notably, the early manifestations were non-specific, making it challenging to distinguish from cerebrovascular diseases, neurodegenerative disorders, and other central nervous system infections. A close examination of the diagnostic trajectory reveals that detailed epidemiological history, serological screening, neuroimaging, and cerebrospinal fluid pathogen analysis played complementary and sequential roles in advancing the diagnosis. Importantly, in the context of atypical neuropsychiatric features, metagenomic next-generation sequencing (mNGS) provided a decisive basis for pathogen confirmation. From this perspective, it follows that for complex cases with non-specific routine findings, establishing a comprehensive diagnostic approach integrating clinical presentation, exposure history, and pathogen evidence remains a critical strategy to improve the identification of neurobrucellosis and optimize patient outcomes.

#### 2.2.1 Differential diagnosis analysis

Neurological brucellosis is an uncommon complication of Brucella infection, with the incidence of nervous system involvement ranging from 1.7% to 4% (3, 9–12). The clinical symptoms of neurological brucellosis are highly variable and often misdiagnosed as other neurological disorders, such as Alzheimer's disease (AD), schizophrenia, severe depression, tuberculous meningitis, or demyelinating spinal cord diseases. These conditions, which typically feature cognitive dysfunction and behavioral changes (3, 11, 13), can be especially challenging to differentiate in elderly patients, where symptoms may overlap with the normal aging process or AD. As such, it is critical to distinguish neurological brucellosis from other neurological infections.

In this case, before brucellosis was definitively established, the patient's presentation—characterized mainly by fever, headache, cognitive impairment, and abnormal psychiatric and behavioral symptoms—prompted consideration of a broad differential diagnosis, including neurodegenerative disorders such as Alzheimer's disease, psychiatric illness, cerebrovascular disease, and central nervous system infections such as tuberculous meningitis or viral encephalitis. Early diagnostic evaluation relied largely on serological testing, particularly the Brucella agglutination test, together with routine laboratory examinations and neuroimaging assessments such as cranial MRI. These findings, however, lacked specificity and therefore carried a substantial risk of misinterpretation. At initial presentation, the patient was seen for recurrent fever, headache, and disturbance of consciousness, and the imaging findings of cerebral infarction and white matter degeneration at one point made cerebrovascular or neurodegenerative disease appear more likely. It was only after a more detailed epidemiological history was obtained, including sheep breeding and exposure, along with positive serological results, that the diagnostic focus gradually shifted toward Brucella infection. On the basis of empirical anti-infective therapy, subsequent cerebrospinal fluid analysis and metagenomic next-generation sequencing (mNGS) ultimately confirmed the diagnosis of neurobrucellosis.

#### 2.2.2 Comparison with previous studies

The most common clinical manifestations of neurological brucellosis include disturbances in consciousness, speech impairments, psychiatric and behavioral changes, cognitive deficits, and headaches (2, 3, 11, 14). This case report focuses on cognitive decline and psychiatric abnormalities in particular. In a cohort study involving 39 patients with neurological brucellosis, Guven et al. identified the most frequent symptoms as headache (76.9%), fever (66.7%), vomiting (43.6%), and meningeal signs (43.6%). Neurological complications included cranial nerve abnormalities (35.9%), spinal cord disease (10.3%), and psychiatric symptoms (10.3%) (3, 12, 15, 16). Research has shown that following Brucella infection, infected mononuclear phagocytes undergo significant transcriptional changes during the adaptation phase. These changes return to baseline after 12 h, when Brucella begins to replicate. Once adapted to the macrophage's internal environment, Brucella can persist intracellularly indefinitely, leading to infection of specific target cells or tissues, including the reticuloendothelial system, endothelial cells, male reproductive organs, fetal lungs, skeletal tissues, trophoblast cells in the placenta, and potentially causing systemic metastasis (6, 17). In clinical practice, a comprehensive epidemiological history and laboratory tests, such as the Brucella agglutination test and cerebrospinal fluid examination, are often crucial for confirming the diagnosis or ruling out other neurological disorders.

Although the incidence of neurological brucellosis is relatively low, it is crucial to strongly consider Brucella infection in regions where it is endemic, particularly in areas with large livestock populations, when patients present with relevant symptoms (18, 19). Research has demonstrated that cases are more prevalent in households raising animals such as camels, sheep, goats, and dairy cattle (20–22). Diagnosing brucellosis in non-endemic areas presents a challenge, as it is a non-specific febrile illness, underscoring the importance of appropriate testing (19). Early diagnosis can significantly aid in preventing disease progression, alleviating symptoms, and enhancing treatment outcomes (23). Another study suggests that Brucella is particularly adept at evading immune responses, spreading throughout the body, and resisting eradication by conventional antibiotics, which complicates its cure (3, 24, 25). Drug resistance remains a critical concern (21). In Ulanqab, Inner Mongolia, Liu et al. studied 85 strains of *Brucella melitensis* isolated from sheep and assessed their resistance to nine antibiotics. All isolates were found to be susceptible to ciprofloxacin, gentamicin, levofloxacin, minocycline, sparfloxacin, doxycycline, and tetracycline. However, 1.0% (1/85) of the isolates showed resistance to rifampin, and 7.0% (6/85) were resistant to co-trimoxazole. Notably, the rifampin-resistant isolate did not exhibit mutations in the *rpoB* gene (2, 6, 26). In this case, the patient's family history of brucellosis and their direct contact with sheep provided valuable diagnostic clues. Fortunately, the patient remained sensitive to rifampin, doxycycline, ceftriaxone, and co-trimoxazole. Given that the clinical presentation of neurological brucellosis often mirrors other neurological disorders, it is essential to proactively conduct Brucella-related tests when such symptoms arise.

#### 2.2.3 Limitations and future prospects

Although this case is, to some extent, representative in terms of its diagnostic and therapeutic course, it still has several limitations, and the diagnostic strategy applied also has certain methodological features that should be interpreted in the appropriate clinical context. First, as a single case report, its generalizability is inherently limited. In addition, the clinical manifestations were non-specific, which created a clear risk of delayed diagnosis, and some key investigations were performed relatively late in the disease course. For example, cerebrospinal fluid examination and pathogen detection, particularly metagenomic next-generation sequencing (mNGS), were not carried out until the illness had already progressed, which may have reduced the opportunity for an earlier and more precise diagnosis. Theoretically, if cerebrospinal fluid analysis and molecular testing had been performed when obvious neurological symptoms first appeared, the underlying cause might have been identified sooner.

From a methodological perspective, however, this case also illustrates certain strengths. A multilayered and integrated diagnostic pathway was adopted: initial screening relied on serological testing, especially the Brucella agglutination test, together with routine laboratory examinations; empirical treatment was then initiated once epidemiological evidence became supportive, while imaging studies were used to assess the extent of the lesions. This combination of clinical evaluation, epidemiological investigation, and laboratory testing is broadly consistent with the general principles of diagnosing infectious diseases.

It should also be noted that follow-up data and long-term prognostic evaluation remain relatively insufficient. Although the patient's symptoms improved by the time of discharge, residual manifestations, such as slowed responsiveness, were still present, and the subsequent therapeutic outcome and risk of relapse require longer observation. This, in turn, suggests that in similar cases, continued follow-up is of considerable importance for assessing treatment efficacy and optimizing management strategies.

With the advancement of molecular biology technologies, metagenomic next-generation sequencing (mNGS) has become an essential tool for diagnosing brain and complex infections (27, 28). mNGS not only enables precise identification of pathogens such as Brucella but also proves invaluable in cases where traditional diagnostic methods fall short of detecting the infection (11, 14, 29). In this case, mNGS played a crucial role in the early diagnosis of neurological brucellosis in cerebrospinal fluid and was instrumental in the differential diagnosis (30). Looking ahead, mNGS holds considerable promise as a standard diagnostic method for neurological brucellosis (11, 31). Treatment of neurological brucellosis typically involves a combination of antimicrobial drugs to ensure the complete eradication of the infection (32, 33). In this case, the patient was treated with a regimen of rifampin, doxycycline, ceftriaxone, and co-trimoxazole. After more than 2 months of treatment, the Brucella agglutination test showed a decrease from 1:200++++ to 1:200+, along with notable improvement in the patient's symptoms. However, given the prolonged nature of the illness, the patient will require ongoing follow-up after discharge to assess the long-term effectiveness of the treatment and to monitor for any potential relapse.

In terms of treatment, combination antimicrobial therapy remains the cornerstone of management for neurobrucellosis. Even so, there is still a lack of sufficiently consistent high-quality evidence regarding the optimal drug regimen, treatment duration, and individualized therapeutic strategies for patients with different clinical subtypes (32). This is particularly relevant in patients with nervous system involvement, in whom the disease course is often prolonged and some cases may be complicated by variable treatment responses, drug-related adverse effects, and the risk of relapse (22, 32). For that reason, future work should ideally rely on multicenter studies with larger sample sizes to stratify patients according to age, mode of onset, and patterns of neurological involvement, thereby allowing treatment protocols to be refined and more detailed systems for efficacy assessment and relapse monitoring to be established.

From the perspective of public health and clinical management, strengthening epidemiological surveillance is equally important. As the epidemiological pattern of brucellosis continues to evolve, sporadic cases may gradually emerge in non-traditional endemic areas, where underrecognition and diagnostic delay are often more likely to occur (33). Continued efforts in regional surveillance, physician training, and case database development may help identify such atypical cases at an earlier stage and, in turn, provide a more reliable basis for evaluating disease burden and optimizing diagnostic and therapeutic strategies.

In summary, while some literature has addressed neurological brucellosis, its varied clinical presentations and lack of specificity continue to present significant challenges for early diagnosis (11, 14). This case underscores the importance of considering neurological brucellosis in patients with neurological symptoms and a relevant epidemiological history. Once the diagnosis is established, it is crucial to take into account factors such as patient adherence to treatment and the potential for drug side effects. Looking ahead, there should be increased focus on epidemiological surveillance, clinical studies, and the advancement of new diagnostic methods for this disease. Moreover, more clinical data is needed to support the development of treatment plans and efficacy evaluations tailored to different age groups and clinical manifestations.

## Data Availability

The original contributions presented in the study are included in the article/Supplementary material, further inquiries can be directed to the corresponding author.
