# Supplementary material for: Case Report: Neurological brucellosis with behavioral abnormalities: a case of Brucella encephalitis with mild cognitive impairment
Source: Front Med (Lausanne). 2026 Apr 15;13:1809313. doi: 10.3389/fmed.2026.1809313 (PMC13124631; doi:10.3389/fmed.2026.1809313)
Supplement: Supplementary file 1 [file Table_1.docx]

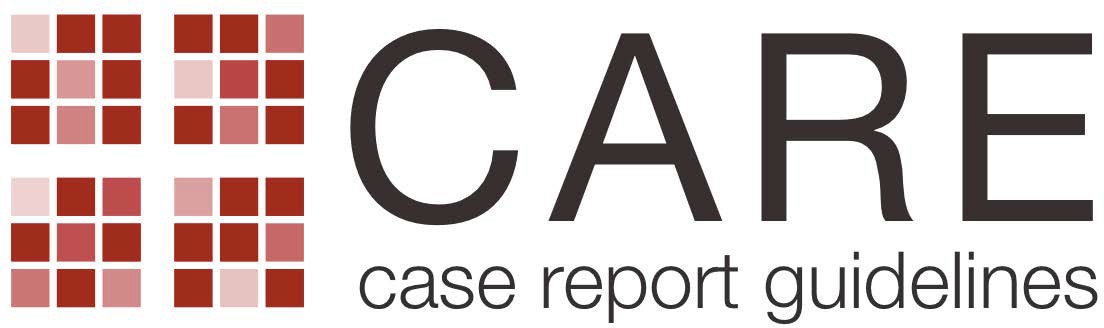
CARE Checklist of information to include when writing a case report
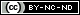


**Topic Item Checklist item description Reported on Line**

**Title 1** The diagnosis or intervention of primary focus followed by the words “case report” . . . . . . . . . . . . . . . . . . Neurological Brucellosis with Behavioral Abnormalities: A Case of Brucella Encephalitis with Mild Cognitive Impairment

**Key Words 2** 2 to 5 key words that identify diagnoses or interventions in this case report, including "case report" Neurological Brucellosis, Abnormal behavior, Encephalitis, Cognitive impairment, Diagnosis, NGS, Case report

**Abstract**

**(no references)**

**3a** Introduction: What is unique about this case and what does it add to the scientific literature? A case of neuropathogenic brucellosis presenting with behavioral abnormalities and mild cognitive impairment is reported, contributing to the diagnosis and treatment of the disease.

**3b** Main symptoms and/or important clinical findings . . . . . . . . . . . . . . . . . . . . . . . . . . . . . . . . . . . . . . . . . . . . . . . . . . . Abnormal behavior with cognitive impairment, poor self-care abilities, inattention, non-fluent aphasia, memory impairment, bilateral resting and action tremors, bradykinesia, and decreased muscle strength.

**3c** The main diagnoses, therapeutic interventions, and outcomes Neurological brucellosis, combination antibiotic therapy, cognitive impairment and behavioral improvement

**3d** Conclusion—What is the main “take-away” lesson(s) from this case? Providing new insights for diagnosing atypical neurogenic brucellosis

**Introduction 4** One or two paragraphs summarizing why this case is unique (**may include references**) Initially, the patient presented with typical brucellosis, consistent with both epidemiological history and ancillary tests. After a period of treatment, the patient reported subjective improvement in symptoms but discontinued medication due to significant drug side effects. Approximately two weeks after discontinuing medication, the patient developed neurological symptoms accompanied by cognitive impairment. Given the patient's advanced age, conditions such as cerebral infarction or Alzheimer's disease were considered. However, after empirical antimicrobial therapy, the patient's symptoms improved. With a preliminary hypothesis, a lumbar puncture was performed alongside next-generation sequencing and cerebrospinal fluid (CSF) analysis, confirming a diagnosis of neuropathic brucellosis. Following aggressive treatment, the patient's condition improved.

**Patient Information 5a** De-identified patient specific information Qingping Wang, male, 70 years old, married, farmer

**5b** Primary concerns and symptoms of the patient Primary concerns: The patient's advanced age, severe neurological symptoms, and cognitive impairment significantly impact daily life and impose substantial financial burdens on the family.

Symptoms: Abnormal behavior with cognitive impairment, poor self-care abilities, inattention, dysarthria, memory impairment, bilateral resting and action tremors, bradykinesia, and decreased muscle strength.

**5c** Medical, family, and psycho-social history including relevant genetic information Epidemiological History: The patient reported assisting relatives who raise sheep at home, with close contact to live sheep and their excrement. His daughter-in-law is currently hospitalized at our institution for “brucellosis.”

Family History: Both parents are deceased; details are therefore unavailable. He has three sisters, all in good health. No other family history of genetic disorders or psychiatric conditions exists.

**5d** Relevant past interventions with outcomes Previous interventions: Treatment with rifampicin, doxycycline hydrochloride, ceftriaxone, and compound sulfamethoxazole for over one month.

Outcome: Both fever and low back pain symptoms improved.

**Clinical Findings**

**Timeline**

**Diagnostic Assessment**

**Therapeutic Intervention**

**Follow-up and Outcomes**

1. Describe significant physical examination (PE) and important clinical findings Physical examination: Inattention, non-fluent aphasia, recall impairment, bilateral resting and action tremors, bradykinesia, and decreased muscle strength.
2. Historical and current information from this episode of care organized as a timeline Nursing History and Course: Three months prior to the onset of neurological symptoms, the patient experienced fever and low back pain. Subsequently diagnosed with brucellosis, the patient was hospitalized for over 20 days, during which symptoms were controlled. After discharge, oral medication was continued for over 10 days. Due to significant drug side effects, the patient discontinued medication for over half a month. Subsequently, the patient developed bradykinesia with behavioral abnormalities and cognitive impairment, leading to presentation at a local hospital for cerebral infarction. Two months ago, the patient returned to our hospital. Empirical antimicrobial therapy was initiated to treat the ongoing brucellosis. Symptoms improved within 2 days. On the third day of admission, guided by a preliminary hypothesis, we performed a lumbar puncture with next-generation sequencing and cerebrospinal fluid (CSF) analysis, confirming neurogenic brucellosis. Following aggressive treatment, the patient's condition has improved.

**8a** Diagnostic testing (such as PE, laboratory testing, imaging, surveys). No clear reports have been identified in the current relevant case reports.

**8b** Diagnostic challenges (such as access to testing, financial, or cultural) The patient exhibits slowed speech and memory impairment. Other neurological conditions cannot be ruled out, such as acute or subacute cerebral infarction, Alzheimer's disease, etc.

**8c** Diagnosis (including other diagnoses considered) The patient is a farmer with limited income and cannot afford certain tests, such as next-generation sequencing.

**8d** Prognosis (such as staging in oncology) where applicable Prognosis: Brucellosis generally has a favorable prognosis, while the prognosis for neurobrucellosis is fair. However, reports indicate that delayed or untreated cases may result in certain sequelae.

**9a** Types of therapeutic intervention (such as pharmacologic, surgical, preventive, self-care) . . . . . . . . . . . . . . . . . Types of Therapeutic Interventions: Pharmacotherapy

**9b** Administration of therapeutic intervention (such as dosage, strength, duration) Management of Therapeutic Interventions: Rifampicin 0.6 g orally once daily Doxycycline Hydrochloride Tablets 0.1 g orally twice daily Compound Sulfamethoxazole 2 Compound orally every 12 hours Ceftriaxone Sodium for Injection 2 g intravenous deep intramuscular injection every 12 hours

**9c** Changes in therapeutic intervention (with rationale) Changes in therapeutic interventions: Promptly remind patients to take oral medications to improve adherence.

**10a** Clinician and patient-assessed outcomes (if available) Assessment Results: Frequency of abnormal behaviors, improvement in cognitive impairment, improvement in language fluency and muscle strength

**10b** Important follow-up diagnostic and other test results 2025-12-03 Cranial MRI: Findings following treatment for inflammation in the right cerebral hemisphere, with softening lesions forming in the affected area; multiple infarcts and lacunar lesions within the brain; partial V-R gap sign in the right basal ganglia region; cerebral atrophy with partial white matter degeneration.

2025-12-04 Brucella Agglutination Test: (+ at 1:200 dilution).

**10c** Intervention adherence and tolerability (How was this assessed?) Intervention compliance and tolerance: First, it relies on the patient's own expectations for recovery from the disease. Then, family members provide supervision and encouragement. Additionally, we conduct regular telephone follow-ups and laboratory tests.

**10d** Adverse and unanticipated events None

**Discussion 11a** A scientific discussion of the strengths AND limitations associated with this case report Strengths: This case represents neurospirochetosis presenting with behavioral abnormalities and mild cognitive impairment as initial symptoms, which has not been previously reported in the current literature.

Limitations: The diagnosis was confirmed based on results from molecular next-generation sequencing (mNGS), combined with the patient's clinical presentation and imaging findings. Traditional cerebrospinal fluid culture did not yield any supportive evidence.

**11b** Discussion of the relevant medical literature **with references** Case Report: Anti-NMDAR encephalitis associated with neurobrucellosis: causality or coexistence? and other literature

**11c** The scientific rationale for any conclusions (including assessment of possible causes) Our conclusion that the patient better meets the scientific criteria for diagnosing neuro-type brucellosis is based on the following evidence: First, the patient's fever and headache align with the typical features of neuro-type brucellosis. Additionally, cranial MRI findings and cerebrospinal fluid mNGS results indicate the presence of Brucella. Second, the onset of symptoms and initiation of treatment occurred in a sequential temporal relationship, with clinical improvement observed post-treatment, supporting a causal association between the two. Third, we ruled out other common causes, including differential diagnoses such as acute or subacute cerebral infarction and Alzheimer's disease. Furthermore, a report by Alderahim et al. describes a 26-year-old Saudi male who developed slowed cognitive processing, behavioral changes, and impaired speech during disease progression, providing additional support for this case conclusion. It should be noted that this remains a single case observation, which cannot definitively establish causality. Additionally, patient-related factors such as financial constraints and dependency may influence the interpretation.

**11d** The primary “take-away” lessons of this case report (without references) in a one paragraph conclusion For patients with a confirmed diagnosis who require long-term oral medication and experience significant side effects, clinicians must closely monitor factors such as patient trust and dependency during follow-up visits to ensure active treatment adherence.

**Patient Perspective 12** The patient should share their perspective in one to two paragraphs on the treatment(s) they received . . . . Like many patients, this individual initially demonstrated limited trust and dependence on our care. However, through our proactive treatment and sustained interaction over time, both the patient and their family developed profound trust in us, expressed deep gratitude, and forged a strong bond of friendship.

**Informed Consent 13** Did the patient give informed consent? Please provide if requested . . . . . . . . . . . . . . . . . . . . . . . . . . . . . . . . . . . . . . **Yes √** **No
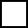
**
